# Case Report: Extensive colonic necrosis and perforation in an HIV patient with syphilis complicated by sepsis

**DOI:** 10.3389/fmed.2025.1687800

**Published:** 2026-01-22

**Authors:** Shouxin Wei, Sijia Yu, Chuan Qian

**Affiliations:** 1 Department of Gastrointestinal Surgery, Suining Central Hospital, Suining, China; 2 Department of General Practice, Suining Central Hospital, Suining, China

**Keywords:** acute abdominal catastrophe, colonic necrosis, HIV infection, perforation, syphilis

## Abstract

**Background:**

‘Soluble’ total colon necrosis is a rare condition with an unclear etiology and few reports on treatment modalities. This case report describes severe intestinal necrosis in an HIV-infected patient, detailing clinical manifestations, diagnostic process, treatment regimen, and outcomes, thereby providing a reference for similar cases in the future.

**Case description:**

The patient is a 41-year-old man diagnosed with HIV 6 years ago, receiving long-term therapy with tenofovir, lamivudine, and efavirenz. He has no history of smoking, alcohol consumption, inflammatory bowel disease, or intestinal tuberculosis, but has had multiple sexual partners. He was admitted with 3 h of abdominal pain; a blood pressure of 108/76 mmHg; a heart rate of 148 bpm, and physical findings of generalized abdominal tenderness, rebound tenderness, muscle rigidity, and diminished bowel sounds. Laboratory results showed elevated procalcitonin (5.15 ng/mL), white blood cells (9.2 × 10^9/L), and C-reactive protein (232.10 mg/L). Abdominal CT revealed thickened walls in the ileocecal region, colon, and rectum, with small bowel dilation, gas, and fluid accumulation, indicating hollow organ perforation and diffuse peritonitis. Emergency laparotomy revealed extensive necrosis of the colon and rectum, with perforation at the hepatic flexure of the colon. A total colectomy, rectal resection, and ileostomy were performed. Postoperative pathology revealed acute and chronic suppurative inflammation with necrosis and perforation. Postoperative sepsis developed, and further examination revealed the presence of syphilis antibodies and liver function impairment. Following treatment with anti-infective, anti-inflammatory, liver-protective, and nutritional support therapies, the patient’s condition improved. On the 9th postoperative day, the patient was transferred to a community hospital for further treatment and was discharged on the 23rd postoperative day.

**Conclusion:**

This case illustrates the rare occurrence of extensive necrosis and perforation of the colon in an HIV-infected patient with co-occurring syphilis. Although the precise role of syphilis in the development of gastrointestinal complications remains uncertain, this case highlights the need for heightened clinical awareness of co-infections in immunocompromised patients.

## 1 Introduction

With the widespread use of combination antiretroviral therapy (cART), the survival rate of HIV-infected individuals has significantly increased (1). Both the virus and long-term drug therapy can induce chronic inflammation, metabolic disorders, endothelial dysfunction, and a hypercoagulable state, thereby increasing the risk of vascular events and acute abdominal conditions (2, 3). Acute mesenteric ischemia has an incidence of approximately 0.09–0.2% in the general population; however, if left untreated, its mortality rate can reach up to 50% (4). HIV-infected individuals are more susceptible to mesenteric ischemia due to hypercoagulability and atherosclerosis. Their clinical manifestations are often masked by non-specific symptoms, resulting in delayed diagnosis (5). In addition to vascular factors, opportunistic infections are significant contributors to gastrointestinal complications in HIV patients. Following the global AIDS epidemic of the 20th century, the spectrum of anal and rectal infections changed, with an increasing incidence of ulcerative colitis caused by cytomegalovirus (CMV) (6). The incidence of syphilis has increased, particularly among men who have sex with men, and it commonly co-infects HIV, often presenting with rectal inflammation or ulcers. A series of reports on syphilis in the lower gastrointestinal tract revealed that over half of co-infected patients with HIV had clinical manifestations often misdiagnosed as inflammatory bowel disease, resulting in delayed diagnosis and treatment, which can lead to complications such as fissures, fistulas, and strictures (7). Additionally, systemic fungal infections, such as mucormycosis, characterized by vascular invasion, involve fungal hyphae penetrating blood vessel walls, leading to thrombosis, interrupted blood supply, and tissue necrosis (8). These opportunistic infections primarily affect immunocompromised hosts and progress rapidly. Previous literature has documented cases of acute abdominal catastrophic events in HIV-infected individuals. For instance, a 45-year-old HIV-positive patient presented with abdominal pain and underwent surgery, during which colonic necrosis was discovered, necessitating a right hemicolectomy. The patient ultimately succumbed to septic shock. The report indicated that HIV and cART-induced hypercoagulable states, along with CMV infection, may jointly trigger mesenteric ischemia and necrosis (9). Another report summarized cases of CMV-associated intestinal perforation, emphasizing that it predominantly occurs in highly immunocompromised individuals with CD4 counts < 50 cells/μL, resulting in an extremely high mortality rate (10). These cases emphasize the importance of considering both vascular ischemia and opportunistic infections as dual factors in HIV-infected patients presenting with an acute abdomen. This case involved a middle-aged male who had been on cART for an extended period. He presented with acute abdominal symptoms and was found to have complete colonic dissolution and necrosis with perforation. Postoperative examination confirmed a concurrent syphilis infection. To explore the rare pathological mechanisms and treatment priorities, this article analyzes the etiology based on relevant literature and summarizes the diagnostic and therapeutic experience.

## 2 Case report

The patient is a 41-year-old man diagnosed with HIV infection 6 years ago and has been receiving treatment with tenofovir, lamivudine, and efavirenz. Six days ago, routine follow-up revealed a CD4 + T-cell count of 278 cells/μL, along with a low CD4/CD8 ratio (0.30). He denied any history of inflammatory bowel disease, intestinal tuberculosis, hypertension, diabetes, or other chronic conditions. Additionally, he reported no history of smoking or alcohol consumption, and prior physical examinations indicated normal liver and kidney function. Three hours prior to admission, the patient suddenly developed persistent pain in the upper and right lower abdomen, which progressively worsened and was accompanied by nausea and vomiting, but without black or bloody stools. He also reported prior unprotected sexual intercourse with multiple partners. Upon admission, the patient exhibited cold and clammy extremities, petechiae on the lower limbs, blood pressure of 108/76 mmHg, a heart rate of 148 beats per min, tachypnea (approximately 35 breaths per min), and a temperature of 37.6 °C. Physical examination revealed marked tenderness throughout the abdomen, rebound tenderness, and significant muscle rigidity. The patient refused further examination, and bowel sounds were notably diminished.

**Figure 1:**
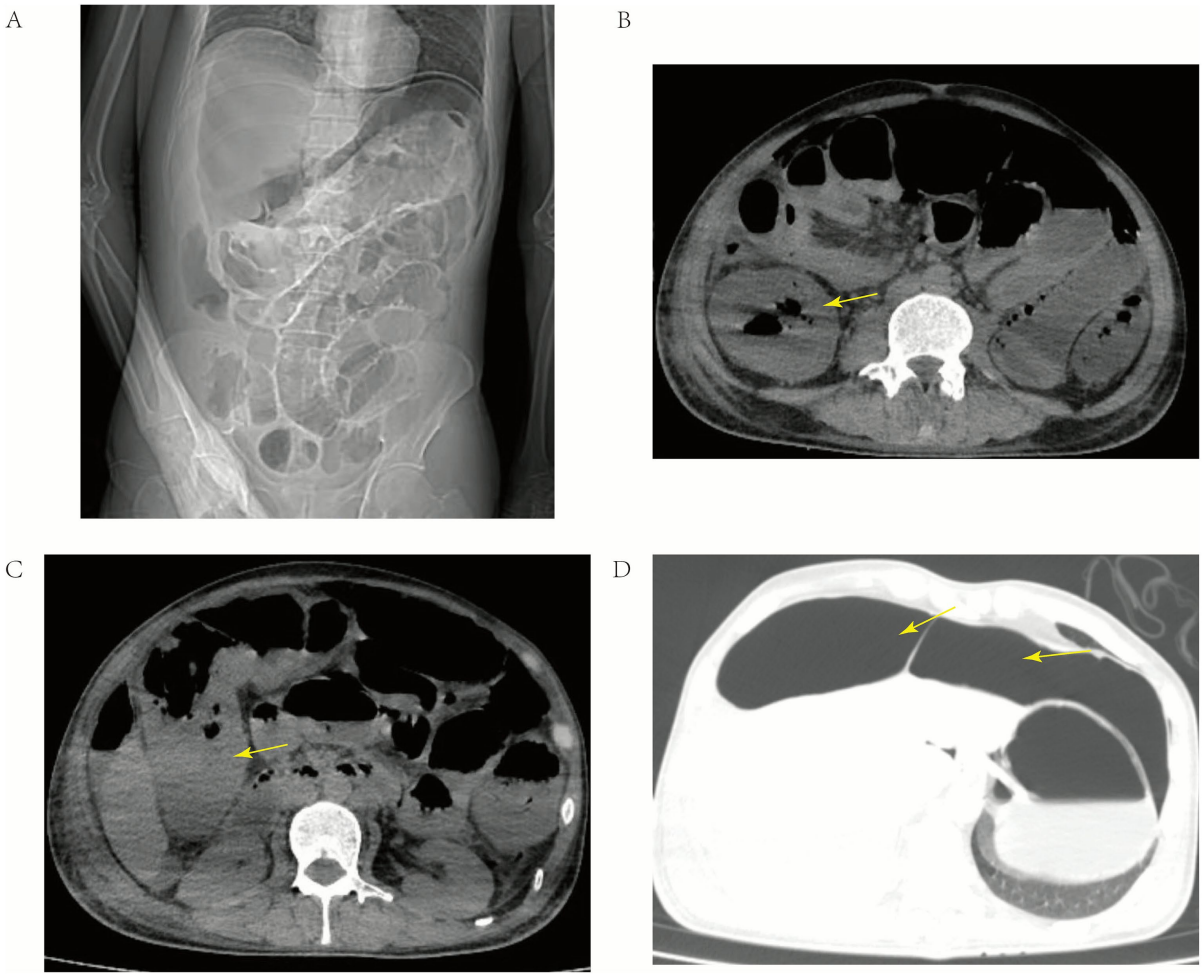
Preoperative abdominal CT images. **(A)** Visible abdominal bowel dilation with a small amount of free gas beneath the diaphragm. **(B)** Marked bowel wall edema, with the thickened ascending colon indicated by the yellow arrow. **(C)** Marked bowel wall edema, with the thickened hepatic flexure of the colon indicated by the yellow arrow. **(D)** Imaging under the lung window, showing free gas in the abdominal cavity, indicated by the yellow arrow.

Laboratory tests upon emergency room admission revealed procalcitonin of 5.15 ng/mL, a white blood cell count of 9.2 × 10^9/L, C-reactive protein of 232.10 mg/L, and interleukin levels consistent with infection. HIV viral load was undetectable, and the CD4 + T lymphocyte count was 250/μL. Syphilis-specific antibody testing was not conducted in the emergency department, while hepatitis B and C viral markers were negative. Abdominal CT imaging revealed diffuse thickening and edema of the ileocecal region, colon, and rectal walls, proximal small bowel dilation with gas and fluid accumulation, free gas in the abdominal cavity, and pelvic fluid, all indicative of hollow viscus perforation and diffuse peritonitis. Immediate treatment was initiated, including for infection control, placement of a nasogastric tube, fluid resuscitation, and blood transfusion preparation, followed by an emergency exploratory laparotomy (Figure 1).

**Figure 2:**
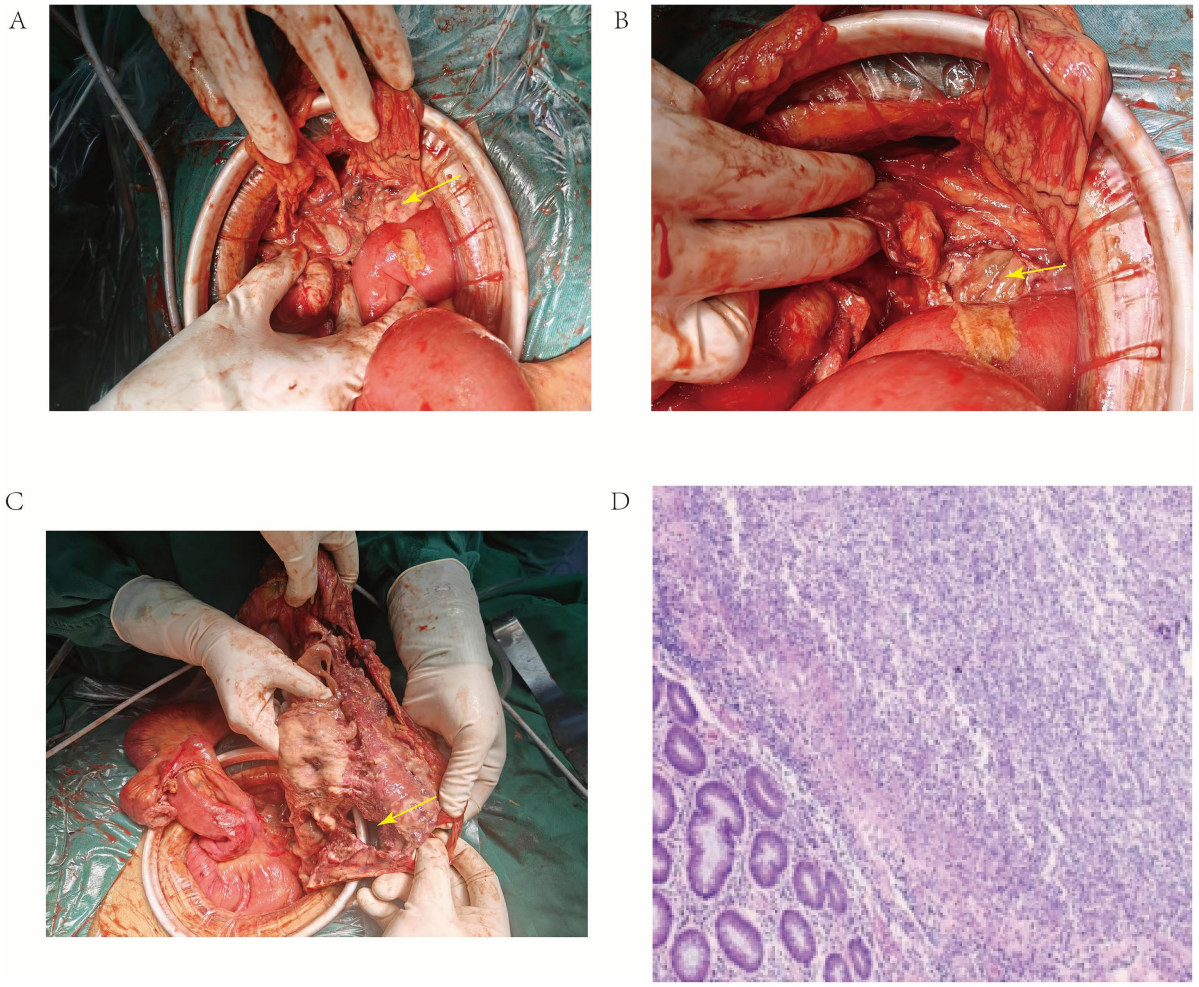
Intraoperative photographs. **(A)** After lavage of the abdominal cavity, the “dissolving” colon is visible, with the transverse colon indicated by the yellow arrow. **(B)** Close-up image shows necrotic and perforated colon at the site indicated by the yellow arrow. **(C)** After freeing and dividing the entire right colon and cecum, necrotic perforated colon with significant thinning, and a large amount of purulent secretion in the bowel lumen, with the hepatic flexure perforation indicated by the yellow arrow. **(D)** Postoperative pathological examination image.

Intraoperative findings included approximately 2000 mL of fecal fluid and a substantial amount of purulent exudate in the abdominal cavity. The mucosa from the ileocecal region to the entire rectum appeared necrotic with a pink ‘dissolving’ appearance, and the intestinal wall was markedly thinned. A perforation, approximately 2 cm in diameter, was identified in the hepatic flexure. No thrombotic lesions were observed intraoperatively. Given the extensive necrosis and poor prognosis, total colectomy with rectal resection and ileostomy was performed. Postoperative pathology confirmed diffuse necrosis and thinning of the entire colon wall, with the thinnest area measuring 0.2 cm. The mucosa was infiltrated by a large number of neutrophils. The final diagnosis included acute and chronic suppurative inflammation with necrosis and perforation, complicated by acute suppurative peritonitis, acute and chronic suppurative appendicitis, and reactive hyperplasia of six paramesenteric lymph nodes (Figure 2). However, despite the suspicion of opportunistic infections in this HIV-infected patient, specialized staining for CMV and *Treponema pallidum* (syphilis) was not performed on the resected tissue.

**Figure 3:**
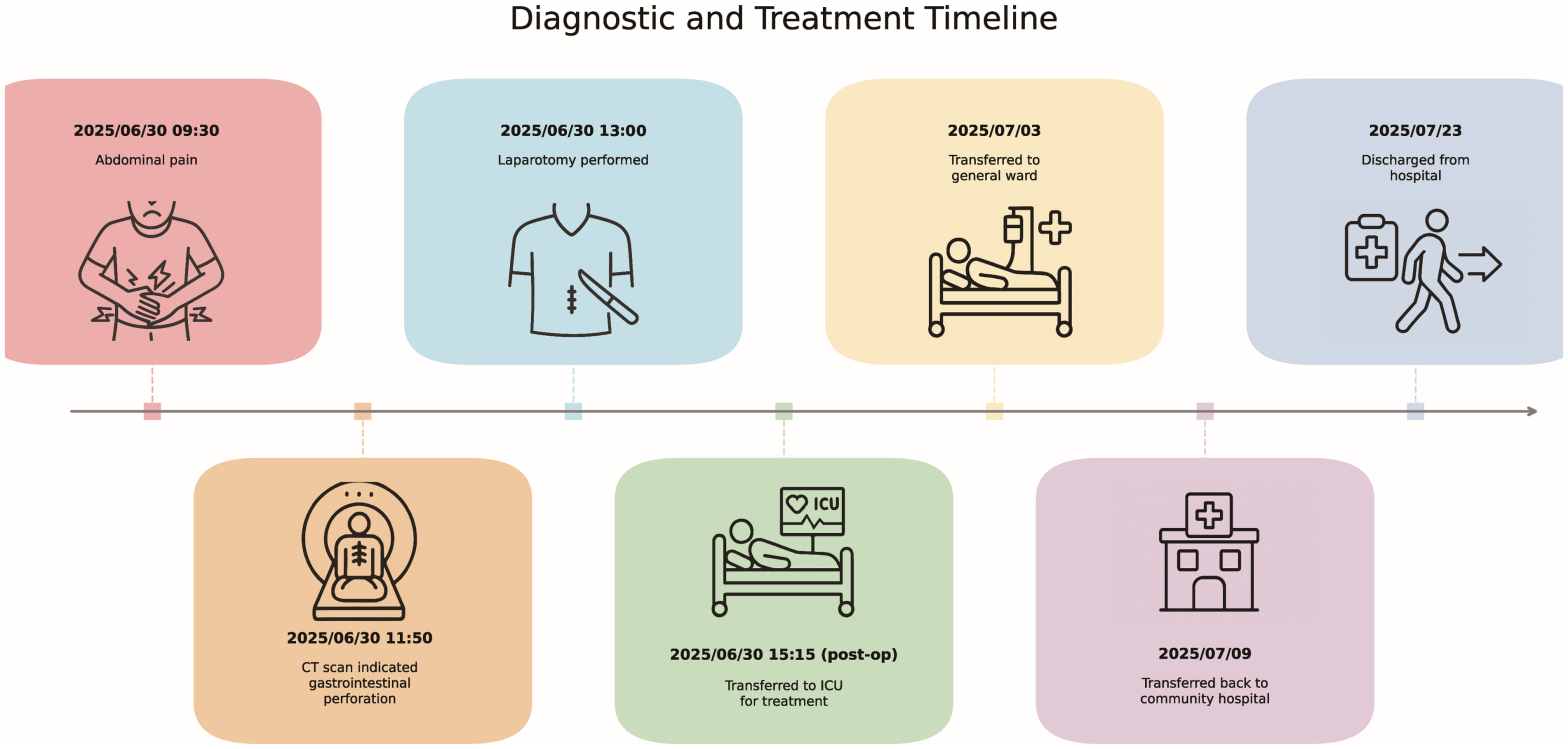
Diagnostic and treatment timeline.

The patient was transferred to the ICU for further management. On the second postoperative day, he developed a high fever (maximum 40 °C), tachycardia, and an increase in procalcitonin to 13.44 ng/mL. IL-6 levels rose to 1986.7 pg./mL, white blood cell count increased to 18.6 × 10^9/L, and CRP was 217 mg/L, fulfilling the criteria for sepsis. Further blood tests revealed positive syphilis-specific antibodies and a positive serum treponemal antibody test, along with concurrent liver function abnormalities (ALT 208 U/L, AST 602 U/L). Following consultation with the Department of Pharmacy, it is recommended to administer potent broad-spectrum antibiotics due to the patient’s extensive intestinal necrosis and compromised immunity, which may predispose him to primary or secondary polymicrobial infections. Based on these findings, magnesium isoglycyrrhizinate was added to the imipramine regimen to protect liver function, along with methylprednisolone and usultiadine for their anti-inflammatory effects. Albumin supplementation and nutritional support were also provided. The patient’s condition gradually improved, with fever resolution and liver function gradually normalizing. By postoperative day 6, procalcitonin had decreased to 8.84 ng/mL, IL-6 levels were 80.1 pg./mL, white blood cell count decreased to 4.4 × 10^9/L, CRP was 233 mg/L, ALT was 111 U/L, and AST was 318 U/L. Both ascites culture and blood culture results indicate Gram-negative bacterial infection. Continue treatment with imipenem for the infection (Supplementary Table S1). On postoperative day 9, the patient was transferred to a community hospital for continued anti-infective therapy and stoma care and was discharged on day 23 (Figure 3).

## 3 Discussion

This patient developed widespread colonic necrosis during combined antiviral therapy. The patient had no history of hypotension, vasoactive drug use, or obvious vascular thrombosis, and no mesenteric vascular occlusion was observed during surgery, suggesting a low likelihood of mechanical ischemia. The nature of the necrosis was ‘dissolving’ rather than the typical purple-black coagulative necrosis, suggesting possible involvement of inflammatory mediators related to infection.

Chronic inflammation, lipid metabolism disorders, and endothelial damage induced by cART can lead to a hypercoagulable state and microthrombus formation, thereby increasing the risk of mesenteric vascular thrombosis (11). Although no obvious arterial thrombus was observed during surgery, widespread necrosis of the intestinal wall and dilation of the proximal intestinal lumen suggested diffuse microcirculatory disorders. Dissolving necrosis may be caused by diffuse ischemia–reperfusion injury induced by microthrombi. Opportunistic infections are common in HIV patients, particularly intestinal infections. Mycosis fungoides typically presents with fungal hyphae invading vascular walls, leading to thrombosis, tissue ischemia, and necrosis; CMV infection can cause intestinal ulcers or even perforation, particularly in immunocompromised states (12, 13). The patient’s CD4 + T-cell count was measured at 278 cells/μL 6 days prior to admission and 250 cells/μL at the time of admission. These values, while reflecting moderate immunosuppression, are still above the threshold commonly associated with severe opportunistic infections such as CMV infection. CMV infection leading to intestinal perforation is classically associated with severe immunosuppression, typically characterized by CD4 + T-cell counts below 50 cells/μL (14, 15). Given the patient’s relatively preserved CD4 count, it seems less likely that CMV was the primary driver of the extensive colonic necrosis observed in this case. This supports the hypothesis that intestinal perforation was more likely a result of cART-induced microvascular disease, which can contribute to vascular dysfunction and ischemia, or severe bacterial and/or syphilitic inflammation.

In this case, a large amount of purulent discharge and liquefactive necrosis was observed intraoperatively. Although fungal organisms were not detected on histopathological examination, the possibility of opportunistic infections causing microangiopathy and necrosis cannot be ruled out. The patient’s serum syphilis antibodies were positive, suggesting active syphilis. Syphilis of the lower gastrointestinal tract is primarily transmitted through anal intercourse and often presents as rectal inflammation or ulcers, which can be easily confused with inflammatory bowel disease (16). Syphilis can cause vasculitis and mucosal ulcers, and in patients receiving antiretroviral therapy, immune reconstitution inflammation syndrome may exacerbate underlying infections, leading to increased tissue damage (17). Therefore, syphilis may have exacerbated intestinal wall inflammation and necrosis in this case. Preoperative laboratory tests showed significantly elevated levels of procalcitonin and interleukin-6, indicating severe bacterial infection. Postoperatively, the patient developed sepsis, and the diffuse necrotic changes in the entire colon were more likely due to infectious necrosis rather than simple vascular gangrene.

This case highlights the importance of promptly and comprehensively evaluating acute abdominal pain in HIV-infected patients, rather than solely considering common gastrointestinal diseases. When imaging reveals diffuse thickening of the ileocecal region and colon wall accompanied by free gas, intestinal perforation or even widespread intestinal necrosis should be suspected, and surgical intervention should be initiated promptly. Postoperatively, monitoring for sepsis and liver function impairment is essential, and appropriate antimicrobial therapy should be administered to address potential opportunistic infections to improve outcomes.

This case report has several advantages. First, it presents a rare and severe case, demonstrating the clinical manifestations of widespread colonic necrosis and perforation in an HIV-infected patient undergoing antiviral therapy. This provides valuable experience for clinicians and enhances their awareness of similar conditions. Additionally, the report provides a detailed diagnostic process, including imaging studies, laboratory tests, and pathological analysis, offering clinicians a comprehensive diagnostic and treatment framework for identifying and managing such complex cases. Timely surgical intervention and postoperative supportive therapy also underscore the critical importance of rapid diagnosis and treatment in similar cases. However, this report also has some limitations. As it is a single case report, the conclusions drawn are to some extent lacking in generalizability and may not be widely applicable to all HIV-infected individuals. While this patient’s postoperative pathology revealed acute and chronic suppurative inflammation with extensive necrosis, we did not perform specialized histological tests for CMV or *Treponema pallidum*, which are known to cause similar gastrointestinal complications in immunocompromised patients, particularly those with HIV infection. Given the absence of histological confirmation for these infections, it remains uncertain to what extent they contributed to the patient’s condition. We acknowledge that this omission limits our ability to draw definitive conclusions regarding the role of these infections and recommend that future cases involve specialized testing for these pathogens when opportunistic infections are suspected. After stabilizing and being discharged, the patient expressed high satisfaction with the timely treatment and surgical intervention. They felt that the care was prompt and effective, and as a token of gratitude, the patient presented a banner to the medical team. This gesture reflects the patient’s appreciation for the care provided and their satisfaction with the outcome. In summary, this case report provides important reference material for clinicians; however, further research and data from multiple centers are needed to validate these observations and explore optimal treatment strategies for similar complex cases.

## 4 Conclusion

This case of HIV infection with widespread colonic necrosis during long-term cART represents a rare and severe gastrointestinal complication. Clinicians must exercise caution when managing similar patients. A comprehensive medical history, timely imaging studies, and prompt surgical intervention are essential for improving survival and recovery in these patients.

## Data Availability

The original contributions presented in the study are included in the article/Supplementary material, further inquiries can be directed to the corresponding author/s.
