# Supplementary material for: Case Report: Extensive colonic necrosis and perforation in an HIV patient with syphilis complicated by sepsis
Source: Front Med (Lausanne). 2026 Jan 22;12:1687800. doi: 10.3389/fmed.2025.1687800 (PMC12872553; doi:10.3389/fmed.2025.1687800)
Supplement: Supplementary file 1 [file Table_1.DOCX]

Supplementary Material

**Supplementary Table 1.** Test results and medical schedule during treatment.

| Test results | Day 1 | Day 2 | Day 4 | Day 7 |
| --- | --- | --- | --- | --- |
| White blood cells (10*10⁹/L) | 9.2 | 18.6 | 4.8 | 4.4 |
| Neutrophils (10*10⁹/L) | 8.32 | 16.85 | 4.28 | 3.45 |
| Haemoglobin (g/L) | 102 | 79 | 50 | 71 |
| Highly sensitive C-reactive protein (mg/L) | 232.1 | 217 | 189 | 233 |
| Serum amyloid protein (mg/L) | No test | >200.00 | >200.00 | 64 |
| Procalcitonin (ng/ml) | 5.15 | 13.44 | 9.03 | 8.84 |
| Interleukin-6 (ng/ml) | 409.3 | 1986.7 | 672.4 | 80.1 |
| Alanine aminotransferase (U/L) | 15 | 208 | 154 | 111 |
| Aspartate aminotransferase (U/L) | 26 | 602 | 437 | 318 |
| Total protein (g/L) | 49.6 | 42.1 | 49.2 | 50.9 |
| Albumin (g/L) | 19.3 | 13.4 | 17.3 | 21.2 |
| Creatinine (umol/L) | 79.5 | 130.7 | 78.2 | 78 |
| Blood potassium (mmol/L) | 2.79 | 5.2 | 2.3 | 3.98 |
| CT report | Diffuse swelling and thickening of the walls of the ileocecal region, colon, and rectum were observed, with blurred surrounding fat planes. The proximal intestinal loops were dilated and fluid-filled, showing air–fluid levels. Multiple foci of free intraperitoneal gas were detected in the abdominal and pelvic cavities, suggesting a possible hollow viscus perforation. Multiple small lymph nodes were seen in the abdominal and pelvic cavities, with thickening of the mesentery and a small amount of ascites. A small right-sided pleural effusion was also noted, with adjacent lung parenchyma showing mild compressive atelectasis. A nasogastric tube was in place. | - | - | - |
| Pathology report | - | - | The submitted specimens of the colon and rectal wall showed acute and chronic suppurative inflammation with necrosis and perforation, secondary acute suppurative peritonitis, and acute and chronic suppurative appendicitis. Six mesenteric lymph nodes exhibited reactive hyperplasia. | - |
| Medical treatment | Piperacillin sodium and tazobactam for infection control, placement of a nasogastric tube, potassium supplementation, and completion of preoperative preparations. Emergency total colectomy and rectal resection with ileostomy performed. Postoperatively transferred to the ICU. | Endotracheal intubation with mechanical ventilation,midazolam and sufentanil for sedation and pain relief, norepinephrine to maintain circulation, esomeprazole for gastric protection, magnesium isoglycyrrhizinate to protect liver function, methylprednisolone and usultrapred for anti-inflammatory effects, changed antibiotics to imipenem and ceftazidime, albumin infusion, and fluid replacement to maintain energy, fluid, and electrolyte balance. | Transferred back to the general ward. Nasal cannula oxygen therapy. Esomeprazole for gastric protection. Magnesium isoglycyrrhizinate to protect liver function. Imipenem and cilastatin sodium for infection control. Blood transfusion. Albumin infusion. Potassium infusion via intravenous pump. Fluid replacement to maintain energy, water, and electrolyte balance. Oral enteral nutrition powder. | Nasal cannula oxygen therapy, esomeprazole for gastric protection, magnesium isoglycyrrhizinate for liver function protection, imipenem and cilastatin sodium for infection control, albumin infusion, fluid replacement to maintain energy and electrolyte balance, and a liquid diet. |
